# Soybean GmPHD-Type Transcription Regulators Improve Stress Tolerance in Transgenic Arabidopsis Plants

**DOI:** 10.1371/journal.pone.0007209

**Published:** 2009-09-30

**Authors:** Wei Wei, Jian Huang, Yu-Jun Hao, Hong-Feng Zou, Hui-Wen Wang, Jing-Yun Zhao, Xue-Yi Liu, Wan-Ke Zhang, Biao Ma, Jin-Song Zhang, Shou-Yi Chen

**Affiliations:** 1 Plant Gene Research Center, National Key Laboratory of Plant Genomics, Institute of Genetics and Developmental Biology, Chinese Academy of Sciences, Beijing, China; 2 Institute of economic crops, Shanxi Academy of Agricultural Sciences, Fenyang, Shanxi, China; Cairo University, Egypt

## Abstract

**Background:**

Soybean [Glycine max (L.) Merr.] is one of the most important crops for oil and protein resource. Improvement of stress tolerance will be beneficial for soybean seed production.

**Principal Findings:**

Six *GmPHD* genes encoding Alfin1-type PHD finger protein were identified and their expressions differentially responded to drought, salt, cold and ABA treatments. The six GmPHDs were nuclear proteins and showed ability to bind the cis-element “GTGGAG”. The N-terminal domain of GmPHD played a major role in DNA binding. Using a protoplast assay system, we find that GmPHD1 to GmPHD5 had transcriptional suppression activity whereas GmPHD6 did not have. In yeast assay, the GmPHD6 can form homodimer and heterodimer with the other GmPHDs except GmPHD2. The N-terminal plus the variable regions but not the PHD-finger is required for the dimerization. Transgenic Arabidopsis plants overexpressing the *GmPHD2* showed salt tolerance when compared with the wild type plants. This tolerance was likely achieved by diminishing the oxidative stress through regulation of downstream genes.

**Significance:**

These results provide important clues for soybean stress tolerance through manipulation of PHD-type transcription regulator.

## Introduction

Drought and high salinity are the major factors to affect plant growth and productivity. These environmental stresses cause the changes of physiological and biochemical processes through alteration of gene expressions. Genes induced by various abiotic stresses are classified into two groups. The products of the first group are effector proteins that protect cell membrane system, hold water, control ion homeostasis and scavenge ROS. These proteins include the key enzymes required for osmoprotectants, LEA proteins, aquaporin proteins, chaperones and detoxification enzymes. The products of the second group are regulatory proteins that control perception of signal, signal transduction and transcriptional regulation of gene expression, including protein kinases, enzymes involved in phoshoinositide metabolism and transcription factors. Several transcription factor families have been found to be induced by drought and salt stresses, such as DREB, ERF, WRKY, MYB, bZIP, and NAC families [1]–[7]. DREB1A and AtMYB2 improved the drought and salt tolerance of transgenic plants when transferred into Arabidopsis [8], [9]. Alfin1, a PHD finger protein, was identified as a salt-induced transcriptional factor and enhanced the stress tolerance by ectopic expression in transgenic plants [10].

The PHD finger was first named from the product of the Arabidopsis HAT3.1 gene in 1993 [11]. After that a number of PHD finger proteins have been identified throughout eukaryotic kingdom. The PHD finger is a conserved Cys4-HisCys3 type zinc finger domain similar to RING finger and LIM domain [12], [13], [14]. Plenty of evidences suggest that the PHD finger proteins are most likely to be chromatin-mediated transcriptional regulators. PHD finger proteins such as transcriptional cofactor P300 and CBP are histone acetyltransferases (HATs) that covalently modify the N-terminal tails of histones [15]. As subunits of histone aceyltransferase or histone deacetylase complexes, PHD finger proteins are required for transcriptional activation or transcriptional repression, such as ING1 [16], Pf1 [17], TIF1 [18] and KAP1 [19]. A PHD finger protein, Alfin1, is characterized as a transcriptional factor that can bind to the promoter of *MsPR2* and enhance the expression of *MsPR2* at the transcriptional level [10], [20]–[22]. Moreover, PHD fingers often occur with SET, Bromo and chromodomains that provide additional evidence for correlation with chromatin [12], [23], [24]. Taken together, there are three possible functions related to chromatin for PHD finger: (1) like other zinc fingers, it might be a DNA or RNA-binding domain; (2) similar to the RING and the LIM domain, it may be a protein-protein interaction domain; (3) it may interact with the flexible histone tails or the central part of the histones [25]–[**27**]. Recent studies have suggested two other functions of PHD finger. The PHD finger of MEKK1 and MIR is suggested to act as E3 ubiquitin ligase [28], [29]. However, two groups argued that the PHD finger of MEKK1 and MIR is more similar to RING domain [30], [31]. Furthermore, the PHD fingers of ING2 and AIRE1 are proposed to be phosphoinositide receptors [32]. But other group did not observe the PIPs binding activity of AIRE1 and there is no more evidences supporting the PHD finger as PIPs receptor [33]. To define the role of PHD finger, researchers tend to believe that PHD fingers in diverse proteins might share the common function. However, some PHD fingers may be different from other PHD fingers in both the sequence similarity and function. In addition, many PHD fingers were identified to interact with specific proteins.

Although the PHD-domain-containing proteins have been extensively studied, the protein functions may not solely depend on the PHD finger, and their roles in plant abiotic stress responses were largely obscure. In this study, we identified six GmPHDs from soybean as a specific set of PHD finger proteins. They are responsive to various abiotic stresses at transcription level. Five of the six proteins had transcriptional suppression activity in plant cells. The N-terminal region of GmPHD was mainly responsible for DNA binding. Overexpression of GmPHD2 enhanced the salt tolerance of transgenic Aarabidopsis, and this may be achieved by scavenging of reactive oxygen species (ROS).

## Results

### Cloning and structural analysis of the *GmPHD* family genes

A gene fragment (256 bp) encoding a PHD finger was identified during cDNA-AFLP analysis using stress-tolerant lines and stress-sensitive lines from the population of the recombined inbred lines derived from the soybean Jindou No. 23 (JD23, drought- and salt-tolerant) and Huibuzhi (HBZ, drought- and salt-sensitive). Expression of the corresponding gene was higher in the stress-tolerant pool than that in the stress-sensitive pool (data not shown). After EST assembly, a full-length gene *GmPHD1* (DQ973812), was obtained, which encoded a PHD finger protein of 253 amino acids. Further searching and assembly of soybean EST sequences revealed five other members of this gene family, namely *GmPHD2* to *GmPHD6* (DQ973807, DG973808, DQ973809, DQ973810, and DQ973811). The *GmPHD3* and *GmPHD6* were partial in 5′- and 3′-end respectively, and their full-length open reading frames were obtained using RACE method.

**Figure 1:**
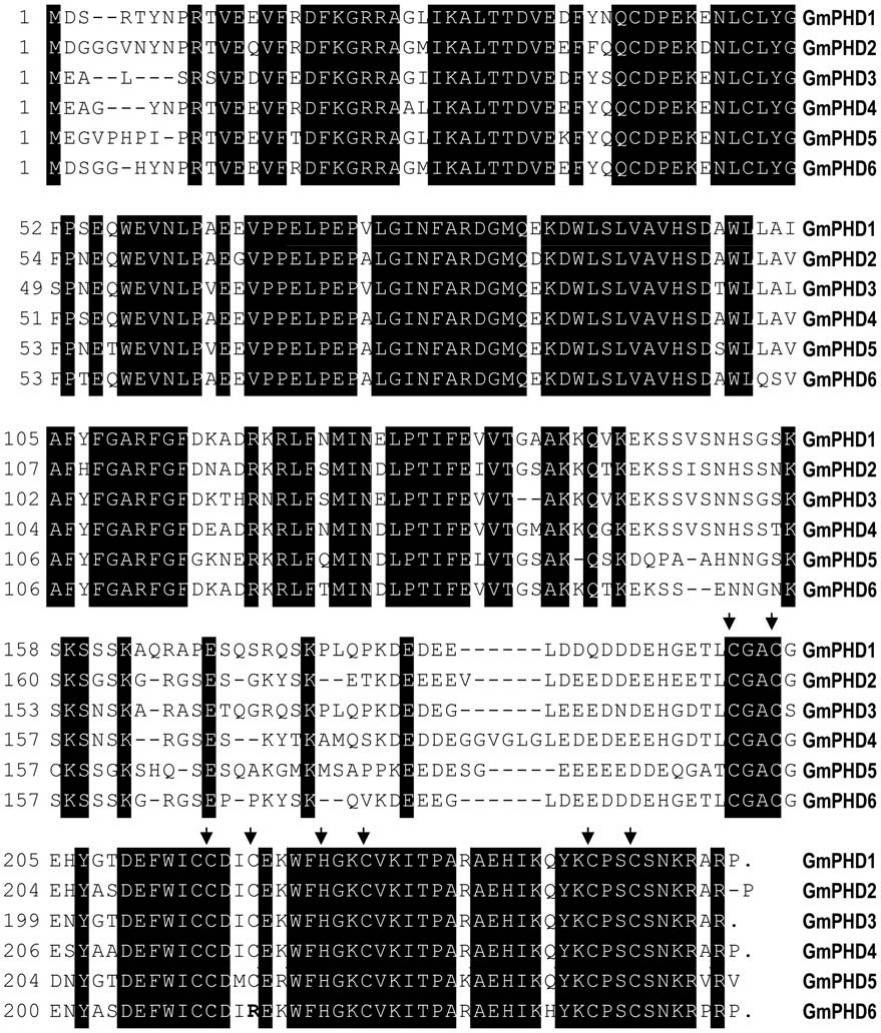
Alignment of the amino acid sequences of the six GmPHD proteins. Identical residues are shaded in black. The C-terminal region is PHD finger and arrows mark the most conserved residues C_4_HC_3_ in the finger.

GmPHDs exhibited 70% to 88% identities with each other. Comparison of the amino acid sequences of these six members of GmPHD family revealed that the N-terminal regions and C-terminal regions were extremely conserved, indicating that these two parts may have significant function (Fig. 1). The C-terminal region is identified as PHD finger, which is a conserved C_4_HC_3_ type zinc finger. However, the fourth cysteine was changed to arginine in GmPHD6. This variation was also found in the homologues of rice (data not shown).

**Figure 2:**
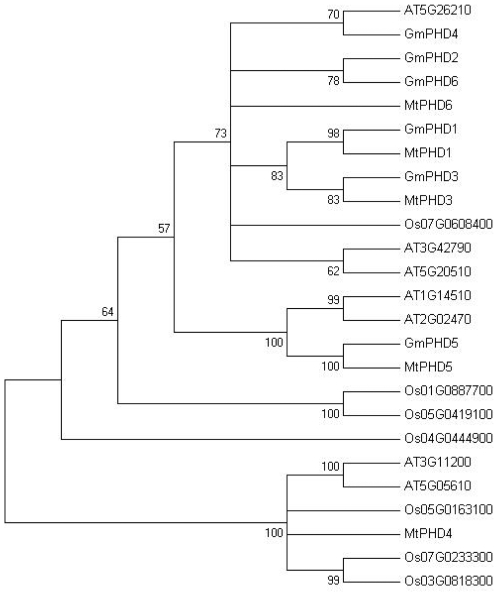
Cluster analysis of the PHD finger proteins from different plants. The analysis was performed by using the MEGA 4.0 program with neighbor joining method and with 1000 replicates. Numbers on the figure are bootstrap values. The sequences are from soybean (GmPHDs), Medicago (MtPHDs), Arabidopsis and rice plants.

The soybean GmPHDs showed 67% to 89% sequence identity to alfalfa Alfin1 (L07291) [10]. Homologues of GmPHDs were also present in many other plant species such as Arabidopsis, rice, Medicago and *Solanum tuberosum*. In *Medicago truncatula*, seven homologues were found and five were full-length sequence termed MtPHD1 to 5 (EF025125, EF025126, EF025127, EF025128, and EF025129). MtPHD5 was almost identical to the Alfin1 [10]. From Arabidopsis and rice databases, seven homologues were also found respectively, including AT1G14510, AT2G02470, AT3G11200, AT3G42790, AT5G05610, AT5G20510, AT5G26210, Os04g0444900, Os05g0163100, Os07g0233300, Os03g0818300, Os05g0419100, Os01g0887700, and Os07g0608400. GmPHDs has an overall identity of 68% to 72% compared to these homologues. The cluster analysis revealed that the GmPHD2, GmPHD4 and GmPHD6 were more closely related whereas GmPHD1 and GmPHD3 grouped with MtPHD1 and MtPHD3 respectively (Fig. 2). The GmPHD5 was clustered with MtPHD5 and may be more divergent when compared with the other GmPHD proteins (Fig. 2).

### Expression profiles of *GmPHDs* under various stresses

**Figure 3:**
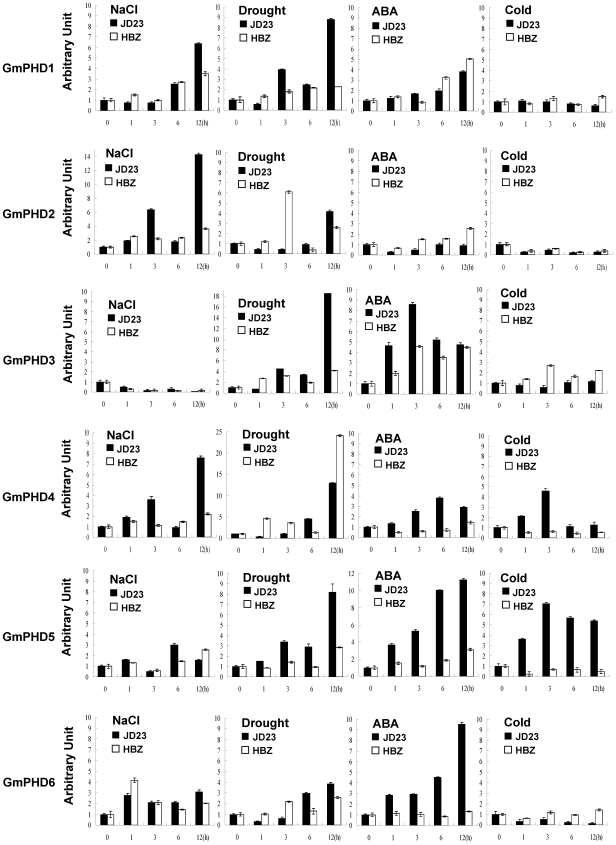
Expression of the six *GmPHD* genes in stress-tolerant cultivar JD23 and stress-sensitive cultivar HBZ under various treatments. Two-week-old soybean seedlings were subjected to treatments with 200 mM NaCl, 100 µM ABA, cold and drought, and total RNA was isolated for real-time quantitative PCR analysis.

Expressions of the six *GmPHDs* were investigated in JD23 and HBZ in response to different treatments, including high NaCl, drought, ABA and cold (Fig. 3). All of the genes were induced in response to drought, but showed differences in responses to the other stresses. One of the genes, *GmPHD4*, was induced in response to all four conditions while the other five genes were induced in response to two or three conditions. Interestingly, the *GmPHD4* and *GmPHD5* were the only genes induced in response to low temperature and in both cases, this was only observed in the more stress-tolerant line JD23. These results indicate that the six *GmPHD* genes were differentially regulated in response to various treatments, and in most cases, the inductions of the *GmPHD* genes were stronger in stress-tolerant JD23 than those in stress-sensitive HBZ.

**Figure 4:**
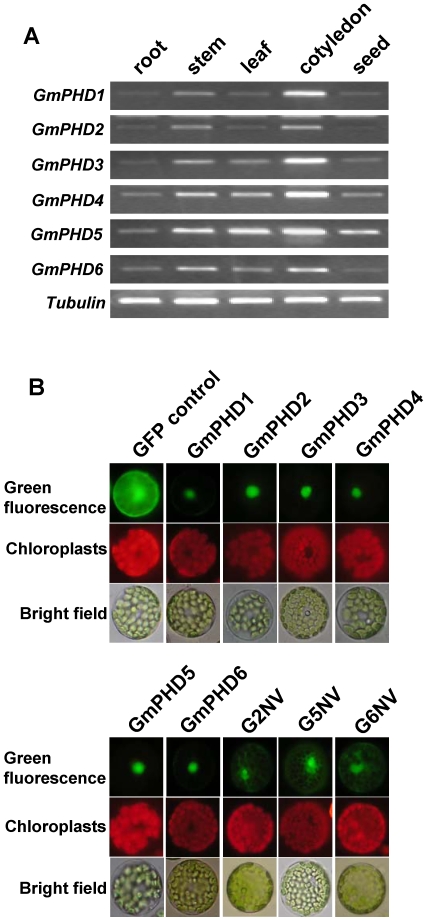
Organ-specific expression and subcellular localization of the GmPHDs. (A) Expression of *GmPHDs* in different organs of soybean plants revealed by RT-PCR. A *Tubulin* fragment was amplified as an internal control. (B) Subcellular localization of GmPHD proteins in Arabidopsis protoplasts as revealed by green fluorescence of. GmPHD-GFP fusions or GFP control. For each panel, the photographs were taken in the dark field for green fluorescence (upper), for red fluorescence indicating chloroplasts (middle), and in the bright light for the morphology of the cells (lower). G2NV: the NV domain of GmPHD2; G5NV: the NV domain of GmPHD5; G6NV: the NV domain of GmPHD6.

The expressions of the six *GmPHDs* were examined in different organs of soybean plants. Fig. 4A showed that all the six genes had relatively higher expression in cotyledons, stems and leaves, but low expression in roots and developing seeds.

### Subcellular localization of GmPHDs

Majority of PHD finger proteins are nuclear proteins but some of them are membrane proteins [34], [35]. Constructs containing the *GmPHDs-GFP* fusion genes in the plasmid pUC18 were generated. The fusion genes and *GFP* control in pUC18 driven by the cauliflower mosaic virus (CaMV) 35S promoter were transformed into Arabidopsis protoplasts, and the protein expression was revealed by the green fluorescence of the fused GFP protein under a fluorescence microscope. All the six GmPHD proteins were targeted to nucleus of the cells, while the control GFP protein was observed in the cytoplasm (Fig. 4B). When the PHD domain (amino acids 198–252) was removed from the GmPHD5, the resulted G5NV truncated protein can be visualized in the cytoplasm although the protein was still abundant in the nuclear region (Fig. 4B). Similarly, when the PHD domain was removed from the GmPHD2 or GmPHD6, the resulted G2NV or G6NV was localized in the cytoplasm and the nuclear region (Fig. 4B). These results indicated that the six GmPHD proteins were nuclear proteins and the PHD domain may play a role in nuclear localization or nuclear retention of the GmPHD proteins.

### Transcriptional regulation activity of GmPHDs

**Figure 5:**
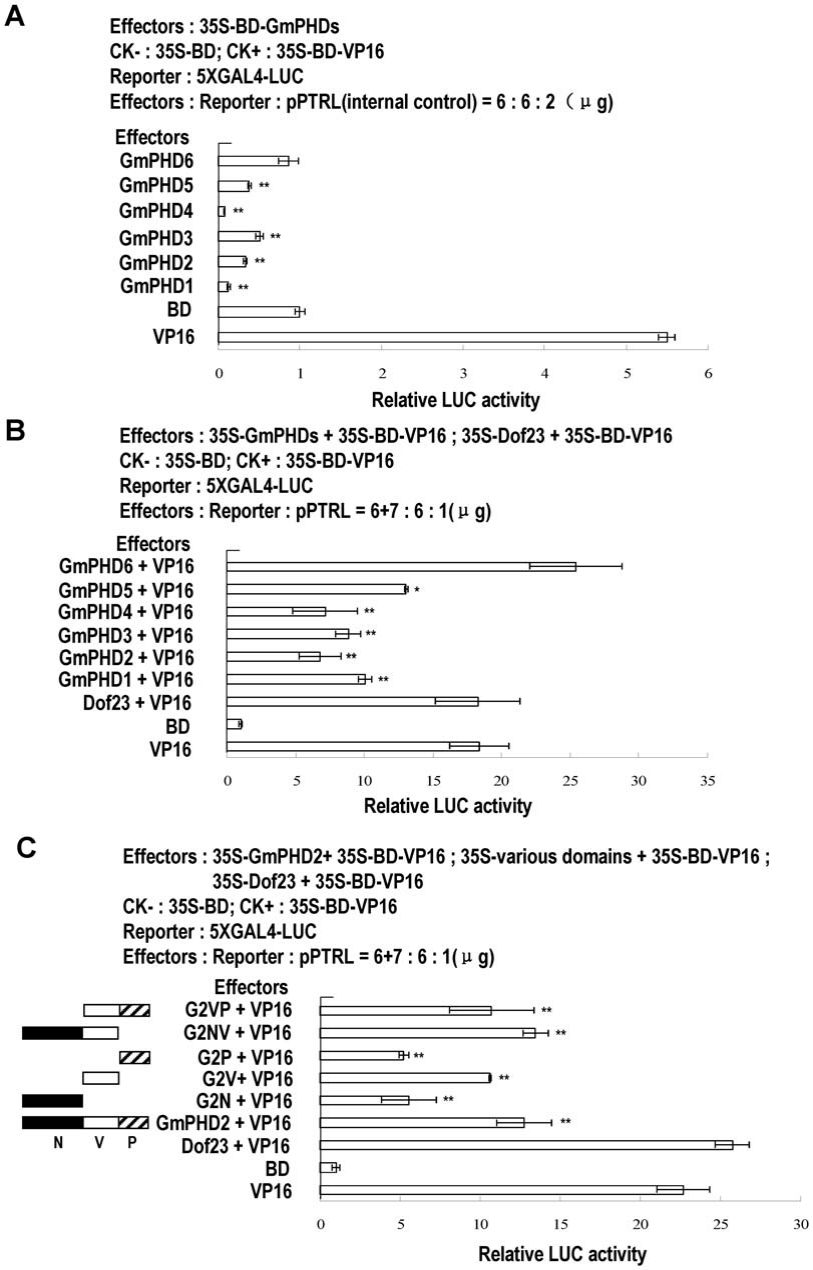
Transcriptional regulation activity of GmPHDs in protoplast assay. (A) Effects of the GmPHDs on reporter gene expression as revealed by relative LUC activity. The GAL4 DNA-binding domain (BD) and VP16 were used as negative and positive controls respectively. “**” indicate highly significant difference (P<0.01) compared to BD value. (B) Effects of the GmPHDs on VP16-mediated LUC gene expression. The Arabiodpsis Dof23 was used as a non-interactive control. (C) Effects of various domains of the GmPHD2 on VP16-mediated LUC gene expression. For (B) and (C), “*” and “**” indicate significant difference (P<0.05 and P<0.01 respectively) compared to VP16 value.

The PHD finger proteins have been reported to have the transcriptional activation activity [36]. We examined the transcriptional activation activity of GmPHDs in protoplast system. As shown in Fig. 5A, among the six proteins compared, five (except the GmPHD6) was found to have inhibitory effect on reporter gene activity when compared to the negative BD control, possibly implying that the five proteins GmPHD1 to GmPHD5 can suppress the transcription of the reporter gene to different degrees. The GmPHD6 appeared not to have such inhibitory activity. To further investigate if the GmPHD proteins have any effect on VP16-mediated transcriptional activation, we included each of the six GmPHD proteins with the positive control VP16 transcription factor in the assay system. Fig. 5B showed that the five proteins GmPHD1 to GmPHD5 had inhibitory effects on VP16-promoted gene expression, suggesting that the five proteins may mainly play roles in transcriptional suppression. On the contrary, the GmPHD6 did not show such ability. A Dof-type transcription factor Dof23 from Arabidopsis did not have significant effect on VP16 transactivation activity.

The GmPHD family members contained conserved N-terminal region, a variable middle part and a conserved C-terminal PHD finger domain. The three regions of the GmPHD2, namely N (N-terminal, amino acids 1 to 117), V (Variable, amino acids 118–196), and PHD (PHD domain, amino acids 197–253) were investigated for their effects on VP16-mediated transcriptional regulation. Fig. 5C showed that the GmPHD2, V domain, NV (amino acids 1 to 196) and VP (amino acids 197 to 253) all had similar inhibitory effects on VP16 transcriptional activation. However, the single N or PHD domain appeared to have stronger roles in transcriptional suppression than the other versions examined, suggesting the importance of the N and PHD domain in transcriptional regulation. Addition of the V to the N domain (G2NV) abrogates the inhibition, suggesting that the regulatory effects may target different molecular aspects as determined by structure of the protein.

### Analysis of the GmPHDs dimerization

**Figure 6:**
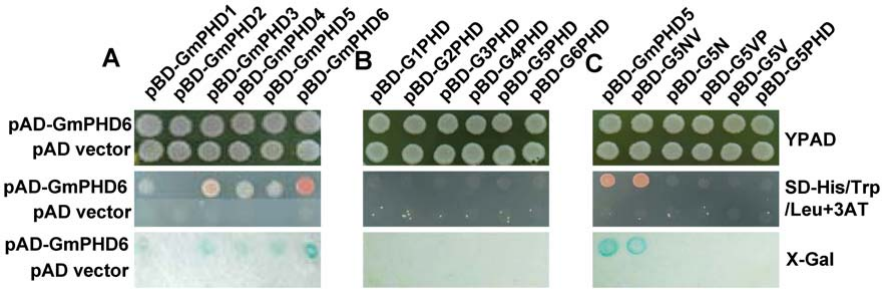
Dimerization ability of the six GmPHD proteins. (A) Dimerization between the GmPHD6 and other GmPHD proteins as revealed by transformant growth on YPAD and SD-His/Trp/Leu plus 3-AT, and by X-gal staining. (B) The PHD finger is not responsible for the dimerization. The yeast transformants containing pAD-GmPHD6 plus each of the PHD finger constructs or pBD vector, were examined for cell growth and X-gal staining. (C) The NV region of the GmPHD5 mediates the interaction between GmPHD5 and GmPHD6. The yeast transformants harboring the pAD-GmPHD6 plus different truncated versions of pBD-GmPHD5 were examined for cell growth and X-gal staining. Truncated proteins: GmPHD5(1–252), G5NV(1–197), G5N(1–115), G5PV(116–252), G5V(116–197), and G5PHD(198–252).

Previous studies have shown that a few PHD finger proteins can form homo- or heterodimers by PHD finger [37]. We then examined if the GmPHDs can dimerize by using the yeast two-hybrid assay. Fig. 6A showed that cells transformed with pAD-GmPHD6 plus pBD-GmPHD1, pBD-GmPHD3, pBD-GmPHD4, pBD-GmPHD5 or pBD-GmPHD6 could grow on SD/His^-^/Trp^-^/Leu^-^ medium with 10 mM 3-amino-1,2,4-triazole (3-AT). Also, the blue color was observed in the X-gal staining with these transformed cells (Fig. 6A). These results indicate that the GmPHD6 can form homodimer and heterodimers with other GmPHDs except GmPHD2. However, other combinations of the GmPHD proteins did not generate any interactions (data not shown). We further examined if the PHD finger is involved in the interaction. Fig. 6B showed that the cells harboring the PHD fingers and the pAD-GmPHD6 could not grow on SD/His^-^/Trp^-^/Leu^-^ medium plus 3-AT and did not have positive X-gal staining, demonstrating that there was no interaction between GmPHD6 and PHD fingers of GmPHDs.

To further determine the interaction domain, we focused on the interaction between GmPHD6 and GmPHD5. Constructs harboring various domains of GmPHD5 in pBD vector were made and transformed into YRG-2 cells with pAD-GmPHD6 or pAD vector (negative control). Fig. 6C showed that only the cells containing pBD-GmPHD5 or pBD-G5NV plus pAD-GmPHD6 grew well and exhibited blue color in the X-gal staining. Removal of the V region from the G5NV protein abolished growth of the corresponding transformants, suggesting that the extremely acidic V region has substantial influence on the interactions between GmPHD proteins. The cells from other combinations could not grow on selection medium and no positive X-gal staining was observed (Fig. 6C). These results indicate that the NV region (amino acids 1 to 197 in GmPHD5) of GmPHDs may be the protein-protein interaction domain that functions in dimerization between GmPHD proteins.

### DNA binding activity of the GmPHDs

**Figure 7:**
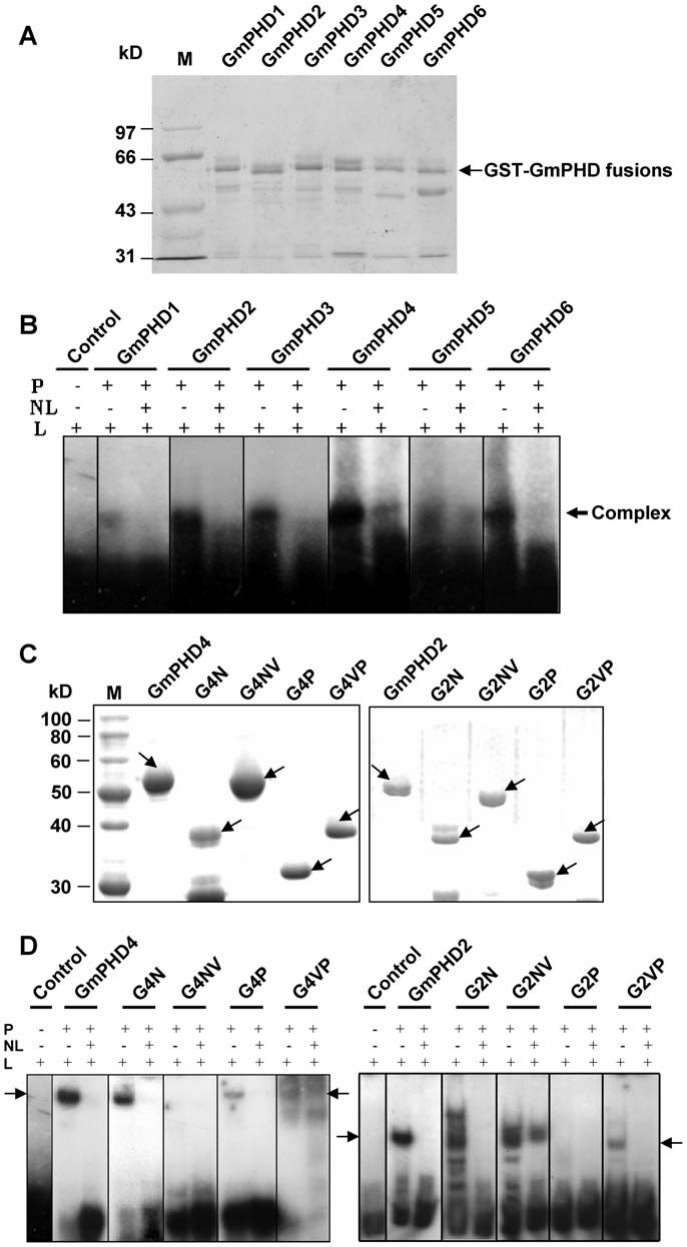
DNA-binding specificity of the GmPHD proteins. (A) Coomassie blue staining of the six GST-GmPHD fusion proteins on SDS/PAGE. Arrow indicates the fusion proteins. Lower bands probably represent the degradation products. (B) Gel shift assay of the six GmPHD proteins. GmPHD proteins (P) were incubated with a radiolabeled probe containing 5 X GTGGAG (L), in the presence (+) or absence (−) of unlabeled probes (NL) in ten-fold excess. Arrow indicates position of the protein/DNA complexes. (C) Coomassie blue staining of various domains of the GmPHD4 and GmPHD2. Arrows indicate the corresponding proteins. (D) Gel shift assay of the GmPHD4, GmPHD2 and their domains. Others are as in (B). Arrows indicate positions of the protein/DNA complexes.

Alfin1, a homologue of GmPHDs from *Medicago sativa*, showed DNA binding activity to the conserved core of GNGGTG or GTGGNG [10]. To identify if the present GmPHDs has any DNA binding activity, we performed gel-shift analysis. Bacterially expressed GST-proteins were isolated and purified (Fig. 7A). Five tandem repeats of the sequence GTGGAG were annealed, labeled and incubated with the six purified GST-GmPHD fusion proteins. All six GmPHDs formed a complex with the labeled GTGGAG and the signal was dramatically decreased by addition of unlabeled DNA probe (Fig. 7B). These results indicate that all the six GmPHDs specifically bind to the GTGGAG element in *vitro*.

To investigate which domain is responsible for the DNA-binding, the GmPHD4 that showed strong DNA-binding activity was used for the analysis. Different domains of the GmPHD4 were expressed (Fig. 7C, left panel) and subjected to DNA-binding assay. Fig. 7D (left panel) showed that the N domain had strong DNA-binding activity whereas the NV domain had no binding activity. The PHD domain had weak DNA-binding ability. The VP domain also had slight DNA-binding in addition to the non-specific binding. To further examine if the roles of different domains in DNA binding are also conserved in other GmPHD proteins, the GmPHD2 and its various domains were expressed (Fig. 7C, right panel) and compared for DNA-binding ability (Fig. 7D, right panel). The N domain of GmPHD2 had strong DNA-binding ability. Presence of the V domain in G2NV did not affect specific DNA-binding but may lead to some non-specific binding. The PHD domain (G2P) showed no DNA-binding while G2VP had weak DNA-binding ability (Fig. 7D, right panel). These results indicate that the N domain had the major ability to bind DNA whereas the PHD domain had weak or no DNA-binding ability. The V domain may have substantial influence on the DNA binding ability of both the N and the PHD domains.

### Transgenic plants overexpressing the *GmPHD2* showed higher salt tolerance

**Figure 8:**
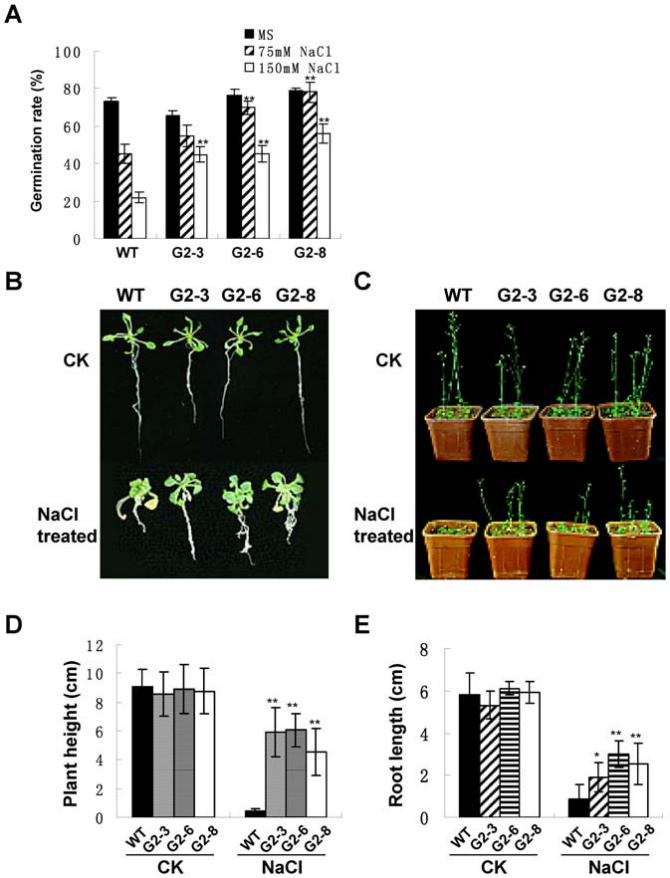
Performance of the *GmPHD2*-transgenic plants under salt stress. (A) Seed germination under salt stress. The seed germination rate of transgenic lines (G2–3, G2–6, G2–8) was calculated 5 d after sowing. Each data point is the means of three replicates and bars indicate SD. (B) Plant growth in NaCl medium. Five-day-old seedlings were treated on plate without (CK, top) or with 150 mM NaCl (NaCl treated, bottom) for two weeks. (C) Recovery of salt-stressed plants in pots. Seedlings treated with 150 mM NaCl (NaCl treated, bottom) or without NaCl (CK, top) were transferred to pots and grown for two weeks under normal conditions. (D) Comparison of plant height after salt stress treatment. Plant heights in (C) were measured. Values are means±SD (n = 54). (E) Comparison of root length after salt stress treatment. Root length of plants in (C) was measured. Values are means±SD (n = 54). For (A), (D) and (E), “*” and “**” indicate significant difference (P<0.05 and P<0.01 respectively) compared to the corresponding WT plants.

**Figure 9:**
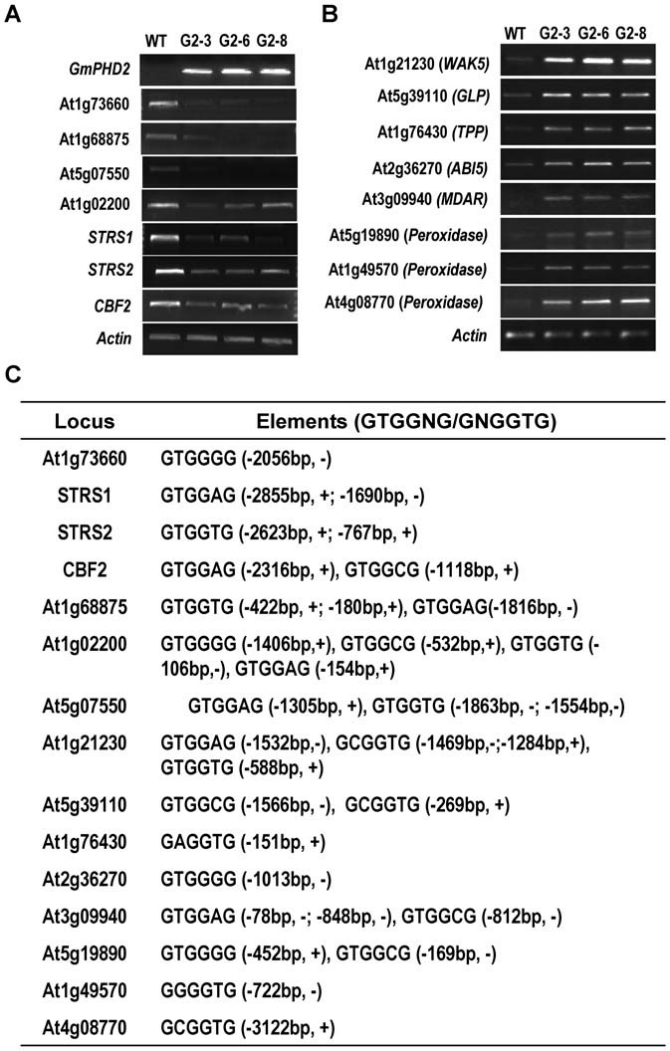
Expression of *GmPHD2*-regulated genes in the transgenic plants. (A) Downregulated gene expression in *GmPHD2*-transgenic plants (G2–3, G2–6, G2–8) revealed by RT-PCR. Two-week-old seedlings were used for RNA isolation. *Actin* was amplified as a control. (B) Upregulated gene expression in *GmPHD2*-transgenic plants. (C) Putative *cis*-DNA elements for GmPHD2 binding in promoter regions of the downregulated and upregulated genes. Numbers indicate the positions upstream the start codon for each gene. “−” indicates that the element was on the antisense strand. “+” indicates that the element was on the sense strand.

Because the *GmPHD* genes were responsive to multiple stresses, we investigated if the GmPHDs are involved in stress responses. The *GmPHD2* was used for further analysis because the encoded protein showed the least homology to the well-studied Alfin1 [10]. We generated the transgenic Arabidopsis plants overexpressing the *GmPHD2* gene under the control of 35S promoter. Three homozygous lines G2–3, G2–6 and G2–8, with higher *GmPHD2* expression (Fig. 8, 9), were analyzed for their performance under salt stress condition. Fig. 8A showed that under normal condition, the germination rate of *GmPHD2*-trangenic seeds was similar to that of the wild type plants. Under NaCl treatment, the germination rate of the transgenic plants was significantly higher than that in the wild type plants. These results indicate that overexpression of *GmPHD2* in *Arabidopsis* enhanced the salt tolerance of the transgenic plants at germination stage.

To evaluate the effects of salt stress on the growth of transgenic plants, five-day-old seedlings of transgenic and wild type plants were transferred onto the plates containing various concentrations of NaCl. After two weeks, we observed severe stressed-phenotype including short roots and compact aerial parts in wild type plants under 150 mM NaCl treatment (Fig. 8B). However, the *GmPHD2*-overexpressing plants had a better growth under the same stress condition. Under normal condition, no significant difference was observed between wild type plants and the transgenic lines (Fig. 8B). The salt-stressed plants in Fig. 8B were further transferred to soil, and their growth status was compared after two weeks. The growth of wild type plants was severely inhibited compared with that of transgenic plants (Fig. 8C). The transgenic plants had higher inflorescences and longer roots than those of wild type plants under salt stress condition (Fig. 8C, D, E). These results indicate that the *GmPHD2* improved the growth of transgenic plants under salt stress.

### GmPHD2-regulated genes in transgenic Arabidopsis plants

Since GmPHD2 has transcriptional suppression activity (Fig. 5), it may inhibit gene expressions. Seven stress-responsive genes were examined for their expressions in *GmPHD2*-transgenic plants. *CBF2/DREB1C* is a negative regulator of *CBF1/DREB1B* and *CBF3/DREB1A* expression, and *cbf2* mutant showed enhanced tolerance to abiotic stresses [38]. *S*T*RS1* and *S*T*RS2* encode DEAD-box RNA helicases and mutations in either genes caused increased tolerance to abiotic stresses [39]. At1g73660 encodes a putative MAPKKK and negatively regulates salt tolerance in Arabidopsis [40]. These four genes were suppressed in the *GmPHD2*-transgenic plants (Fig. 9A). Three other genes At1g68875, At5g07550 and At1g02200 were also inhibited in the transgenic lines (Fig. 9A). At1g68875 encoded a protein of unknown function; At5g07550 encoded a glycine-rich protein, and At1g02200 encoded a putative fatty acid hydrolase with two transmembrane domains.

Eight other genes had higher expression in the transgenic plants in comparison with their expressions in wild type plants (Fig. 9B). These genes included At1g21230 (*WA*K*5*) encoding a wall-associated protein kinase, At5g39110 (*GLP*) encoding a germin-like protein, At1g76430 (T*PP*) encoding a phosphate transporter family protein, At2g36270 (*ABI5*) encoding an ABA-responsive basic leucine zipper transcription factor, At3g09940 (*MDAR*) encoding a putative monodehydroascorbate reductase, three peroxidase genes At5g19890, At1g49570 and At4g08770. The *GmPHD2* gene was also apparently enhanced in the three transgenic lines. These analyses reveal that the GmPHD2 may improve salt tolerance through affecting stress signal transduction and by scavenging ROS.

Because the GmPHD proteins can bind the GTGGAG element, we then examined if the element or its similarities were present in the promoter region of the regulated genes. Fig. 9C showed that in the promoter regions of both the downregulated and upregulated genes, one to four elements were identified. Among the elements from promoter regions of the downregulated genes, the consensus element sequence GTGG(A6/T7/G2/C2)G was found. For the upregulated genes, two consensus element sequence GTGG(A3/T1/G2/C3)G and G(A1/G1/C4)GGTG were identified in their promoter regions (Fig. 9C). These elements may be directly or indirectly involved in *GmPHD2*-regulated gene expression. Considering that the GmPHD2 has transcriptional repression activity, it may bind to the elements and then suppress gene expressions. However, whether the GmPHD2 can bind to the elements from the downregulated genes needs to be further studied.

### Analysis of the oxidative stress tolerance in *GmPHD2*-transgenic plants

**Figure 10:**
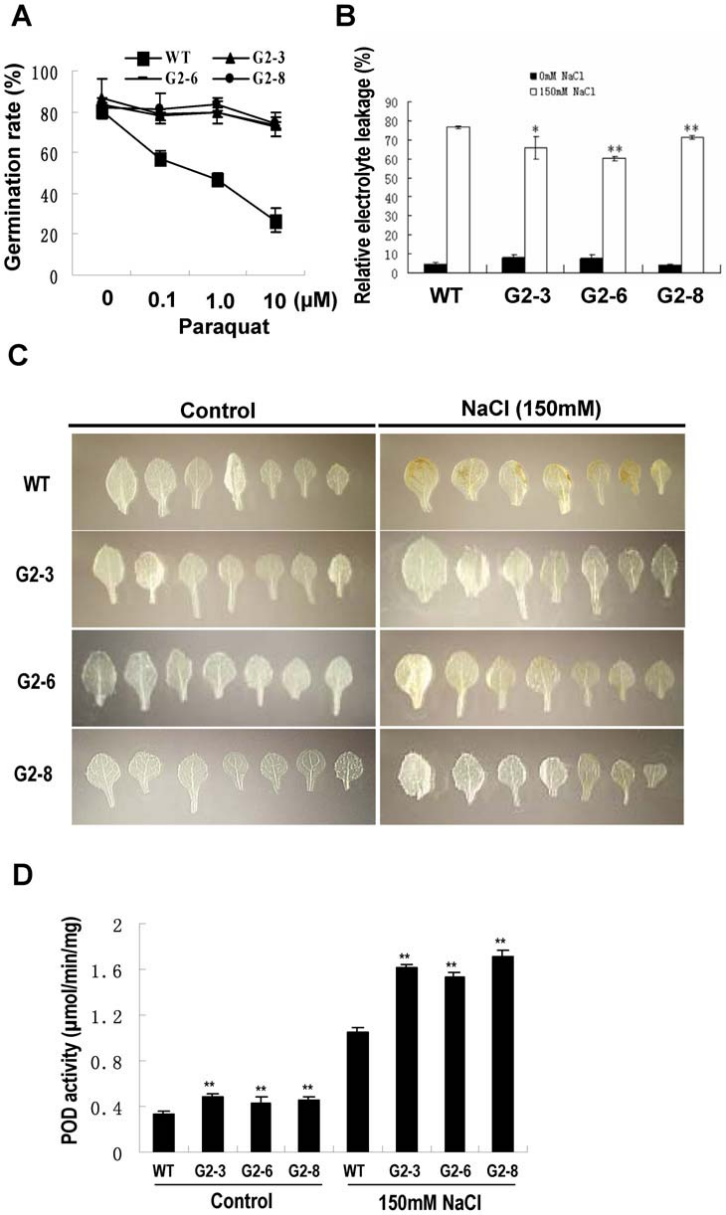
Oxidative stress tolerance of the *GmPHD2*-transgenic plants. (A) Seed germination under paraquat treatment. The germination rate was calculated 5 d after sowing. Values are means±SD (n = 3, each has 80–100 seeds). (B) Electrolyte leakage in salt-stressed plants. Ten-day-old seedlings were subjected to 150 mM NaCl stress for 5 d on plate. Values are means±SD (n = 4, each has four seedlings). (C) Detection of hydrogen peroxide production in plants. Seedlings in (B) were used to detect the H_2_O_2_ levels in leaves. H_2_O_2_ levels were revealed with 3,3′-diaminobenzidine (DAB). Brown color indicates generation of hygrogen peroxide. (D) Peroxidase (POD) activity in salt-stressed plants. Seedlings in (B) were used. Values are means of three replicates and bars indicate SD. For (B) and (D), “*” indicates significant difference (P<0.05) compared to WT. “**” indicate highly significant difference (P<0.01) compared to WT.

Because genes relating to ROS scavenging were identified, we investigated if the transgenic plants overexpressing the *GmPHD2* can tolerate the oxidative stress. Fig. 10A showed that the germination rate of wild type plants was dramatically decreased from ∼80% to ∼23% with the treatments of increasing concentrations of paraquat. However, the germination rates of the transgenic plants were only slightly influenced by the paraquat treatments (Fig. 10A). These results indicate that the seed germination process of the *GmPHD2*-transgenic plants is more tolerant to oxidative stress than that of the wild type plants.

Salt stress usually caused cell membrane damage and resulted in electrolyte leakage. Both the transgenic plants and wild type plants showed higher relative electrolyte leakage under salt stress in comparison with the untreated plants (Fig. 10B). However, the transgenic plants had lower electrolyte leakage than the wild type plants under salt stress. These results indicate that the transgenic plants overexpressing the *GmPHD2* are more tolerant to salt stress than the wild type plants.

The GmPHD2 may confer salt tolerance in the transgenic plants through regulation of oxidative stress. We then examined if the hydrogen peroxide level was changed in the plants under salt stress. Fig. 10C showed that after salt stress, the three transgenic lines showed no brown color whereas the wild type plants showed brown color, the positive response of DAB staining. These results indicate that the wild type plants have more H_2_O_2_ than the transgenic plants, suggesting that the transgenic plants are more tolerant to salt stress possibly through inhibition of H_2_O_2_ accumulation.

Higher expressions of peroxidase genes (Fig. 9) may result in higher peroxidase (POD) activity. Fig. 10D showed that the POD activity in *GmPHD2*-overexpressing plants were 30% to 45% higher than that in wild type plants under normal growth condition. After salt treatment, the POD activity of the transgenic plants and wild type plants all increased at least 3 folds, and the increase of POD activity in transgenic plants was higher than that in wild type plants. These results indicate that GmPHD2 enhanced the POD activity in transgenic plants and this may contribute to the salt tolerance by scavenging hydrogen peroxide.

## Discussion

The present study identified six GmPHD proteins from soybean plants. These proteins shared high identity and belonged to a small family with the PHD finger in the C-terminal end. The transcriptional regulatory activity, DNA binding ability and nuclear localization were revealed for these proteins, indicating that the GmPHD proteins represent novel transcription regulators. The roles of one of these GmPHD proteins, GmPHD2, were investigated through transgenic approach and we find that the GmPHD2 improved stress tolerance in plants.

Among the six proteins, the GmPHD1 to GmPHD5 had transcriptional suppression activity in plant protoplast assay whereas the GmPHD6 did not have such ability, suggesting their different roles in transcriptional regulation. The suppression activity of the five GmPHDs may depend on the presence of the V domain as judged from the GmPHD2 analysis (Fig. 5C). The V domain had similar suppression ability as the whole GmPHD2 did. Removal of the V domain disclosed the strong inhibitory effects of the N or PHD domain, suggesting that the V domain may have a regulatory role for the function of the N and/or PHD domain during transcription.

Three domains can be defined for the six GmPHDs as exemplified in GmPHD2. These included the N domain in the N-terminal conserved region (amino acids 1 to 117), the PHD domain in the C-terminal conserved region with a PHD finger (amino acids 197–253), and the V domain in the variable region between the N and the PHD domains (amino acids 118–196). In addition to transcriptional regulation, roles of these domains in protein dimerization, localization and DNA-binding were also studied. PHD finger has been regarded as a protein-protein interaction domain [41], [42]. The present GmPHD6 can form homodimer. It can also form heterodimers with GmPHD1, GmPHD3, GmPHD4 and GmPHD5. However, these interactions were not mediated by the PHD finger, but rather by the NV region as in the case of GmPHD5 (amino acids 1 to 197). This fact indicates that the PHD fingers in different proteins may have different roles. The possibility that the PHD finger of GmPHDs may interact with other unknown proteins cannot be excluded. Recently, the PHD fingers of the GmPHD/Alfin-like proteins from Arabidopsis have been found to bind to histone post-translational modifications H3K4me3/2 [43]. Another PHD-containing protein ORC1, the large subunit of the origin recognition complex involved in defining origins of DNA replication, can bind to H3K4me3 with its PHD domain and regulate transcription [44]. Therefore the GmPHD proteins may interact with H3K4me3/2 via the PHD domain and form dimers through the NV region. In addition to the roles in interactions, the PHD domains also play some roles in nuclear localization or retention because removal of this domain in GmPHD2, GmPHD5 and GmPHD6 led to cytoplasmic distribution of the protein (Fig. 4).

The six GmPHD proteins showed high identity to the Alfin1 from alfalfa, which also has a PHD finger at the C-terminal end [10]. Further comparison revealed that the GmPHD5 had highest identity (89%) with the Alfin1, suggesting that the GmPHD5 may be an orthologue of Alfin1 in soybean. The other five GmPHDs may be paralogues of the Alfin1. Alfin1 has been found to enhance the MsPRP2 gene expression by binding to the element GTGGNG [10], [20]. However, five out of the six GmPHDs had transcriptional suppression activity in protoplast assay system. This difference may reflect the divergence of the transcriptional regulatory mechanism between the Alfin1 and the GmPHDs. It is possible that the GmPHD proteins may first suppress gene expression and then indirectly affect expressions of other genes.

The PHD finger has been proposed to bind to DNA or RNA as many other zinc fingers do [11], [12]. However, from the solution structure of the PHD finger from KAP-1, no structural features typical of DNA binding proteins are observed [45]. These studies imply that the DNA-binding ability is equivocal for the PHD fingers in different proteins. Despite the discrepancy, the Alfinl and the present six GmPHD proteins all showed specific DNA binding ability [20]. However, further domain analysis of the GmPHD4 and GmPHD2 disclosed that the N domain had strong DNA binding ability whereas the PHD domain showed no or only slight DNA-binding activity (Fig. 7D). These results suggest that the N domain but not the PHD domain was mainly responsible for DNA binding. It is interesting to find that presence of the V domain has some effects on DNA-binding. In the case of GmPHD4, the V domain plays an inhibitory role on DNA-binding activity of the N domain, whereas in GmPHD2, the V domain promotes DNA-binding in the presence of PHD domain (Fig. 7D). This phenomenon, together with the roles of the V domain in regulation of the N and/or PHD-mediated transcriptional suppression (Fig. 5C), suggests that a specific regulatory mechanism is existed for GmPHD/Alfin1-type transcription regulators. It is possible that the PHD domain of this type of proteins interacts with histone for chromatin regulation whereas N domain binds to DNA. The two coordinate reactions may thus lead to transcriptional suppression, with the regulation from V domain and the NV-mediated dimerization. It should be mentioned that although all the six GmPHD proteins are highly conserved, each one may also have specificities in terms of gene expression (Fig. 3, 4), transcriptional regulation (Fig. 5), dimerization (Fig. 6A), and DNA binding ability (Fig. 7). The V domain may determine the specificity of each protein in regulation of these processes. However, how these are realized requires further investigation.

Eighty-three canonical PHD finger proteins have been identified in Arabidopsis [43]. Only several proteins containing the PHD finger domain have been studied in plants. However, except the conserved C4HC3 residues, other sequences in the PHD domains are divergent. The functions of these proteins are also different, ranging from regulation of anther development and male meiosis [46]–[48] to regulation of vernalization and flowering [49]–[52], disease resistance [53], apical meristem maintenance [54], specification of vasculature and primary root meristem [55], and embryogenesis and sister-chromatid cohesion [56]. Unlike the PHD-containing proteins above, the present GmPHDs shared high homology only with *Alfin1* from *Medicago sativa.* The *Alfin1* can be induced by salt stress and enhance salt tolerance in the transgenic plants [20]–[21]. The present six *GmPHD* genes were differentially expressed in drought- and salt-tolerant JD23 and drought- and salt-sensitive HBZ in response to salt, drought, cold and ABA treatment, indicating that this subset of genes may have specific roles in multiple stress responses. In most cases, these genes were induced to a higher intensity in the tolerant JD23 cultivar than that in the sensitive HBZ, suggesting that the genes may contribute to the stress tolerance of the JD23 cultivar. Different from the specific *Alfin1* expression in roots, the six *GmPHD* genes were expressed in multiple organs. We selected the GmPHD2 for transgenic analysis because this protein had the least homology (67%) with the Alfin1. Overexpression of the *GmPHD2* improved salt tolerance of the transgenic plants, indicating that proteins with transcriptional repression can also confer stress tolerance.

The GmPHD2 may confer salt tolerance through control of ROS signaling and ROS scavenging. ROS scavengers have been reported to eliminate the cytotoxic effects of ROS under different stresses [57], [58]. Consistently, the transgenic plants overexpressing the *GmPHD2* were more tolerant to oxidative stress, and had higher levels of POD activity and lower levels of hydrogen peroxide production under salt stress. It is likely that the GmPHD2 confers salt tolerance at least partially by diminishing oxidative stress. Other possibility may also exist due to the fact that many other genes, e.g. ABA signaling gene *ABI5*, were regulated by the GmPHD2 protein. The GmPHD2-regulated gene expression seemed to be different from that regulated by the Alfin1. The Alfin1 has been found to regulate the *MsPRP* gene expression by binding to the cis-element in the promoter region of this gene [10]. It is therefore possible that each PHD-type transcriptional regulator may contribute to the salt tolerance through upregulation of a specific subset of genes. It should be noted that although the GmPHD2 had transcriptional suppression activity, it still can enhance downstream gene expressions. This may be achieved through indirect regulation or via protein interactions. Several genes including *CBF2*, *S*T*RS1*, *S*T*RS2*, and At1g73660 were also down-regulated in *GmPHD2*-transgenic plants (Fig. 9A). These genes are negative regulators of stress tolerance [38]–[40] and may be the direct target of the GmPHD2.

In soybean plants, we have identified six GmPHD proteins. In other plants examined, similar number of genes was found. Because their differential expression patterns in response to various stresses and different mechanisms for transcriptional regulation, it is possible that each *GmPHD* gene has specificity in regulation of stress responses. However, these genes may also generate coordinate responses for stress tolerance through protein interaction or transcriptional regulation within this small gene family. Further investigation should reveal such possibilities and improve our understanding of the functions of this gene family in regulation for a variety of stress responses.

## Materials and Methods

### Plant materials and treatments

The soybean population of recombinant inbred lines derived from Jindou23 (JD23, salt- and drought-tolerant variety) and Huibuzhi (HBZ, salt- and drought-sensitive variety) were used. Two-week-old seedlings from twenty-four salt and drought-tolerant lines and 24 salt and drought-sensitive lines were placed on Whatman filter paper at 23°C and with 60% humidity for dehydration. After 1 h and 3 h, one leaf from each seedling was harvested and combined for RNA isolation to construct the stress-tolerant and stress-sensitive RNA pool for cDNA-AFLP. The seedlings were immersed with the roots in 200 mM NaCl or 100 µM ABA and maintained for the indicated times. For cold treatment, seedlings were placed at 0°C. For drought treatment, seedlings were placed on Whatman filter paper at 23°C and with 60% humidity. Roots, cotyledons, stems, leaves, and ten-day-old developing seeds from soybean were collected for RNA analysis.

### Gene cloning and RT-PCR analysis

The cDNA-AFLP was conducted as described [59]. Based on the obtained sequence encoding a PHD finger domain, six *GmPHD* genes were identified and cloned by RT-PCR or RACE. Homologous genes from Medicago truncatula, rice and Arabidopsis were also identified. Cluster analysis was conducted using the MEGA 4.0 program.

Stress-responsive genes were examined by RT-PCR with primers as follows: for At1g21230, 5′-gtaggtagaaacatatgtgg-3′ and 5′-GTGTTCCCATGTAAGCGAAG-3′; for AT5G39110, 5′-GATCCAAGTCCACTTCAAGAC-3′ and 5′-CAACATTGACGTCTAACTG-3′; For AT1G76430, 5′-GCTCCTTTGGTTGTGGCTTCT-3′ and 5′-CTAGGAACCAATTGGCTGAGGC-3′; For AT2G36270, 5′-CAACAAGCAGCAGCAGCTGCAG-3′ and 5′-GGATTAGGTTTAGGATTAGTGGG-3′; For AT3G09940, 5′-GTTTGTGCTGGAACTGGAG-3′ and 5′-CAGTACAGATTCTCCAACG-3′; For AT5G19890, 5′-CTTGTGCTGATATCCTCACTTT-3′ and 5′-GTGATCATTCTGATACACACGA-3′; For AT1G49570, 5′-GTTGGAGAATATAACAGCCAAG-3′ and 5′-CCATTACACACAAACGTAACAC-3′; For AT4G08770, 5′-GGAAACCAGAGTGTATTGGTAG-3′ and 5′-GTGATCATTCTGATACACACGA-3′; For At1g73660, 5′-AGAATTTGGGAGATGGAGTGG3′ and 5′-CCTTACCAATTCACTATTCAC-3′; For CBF2, 5′-ATGTTTGGCTCCGATTACG-3′ and 5′-ATAGCTCCATAAGGACACGT-3′; For STRS1, 5′-ATGGCTGGACAAAAGCAAGA-3′ and 5′-CATATCAAGCATTCGATCTGC-3′; For STRS2, 5′-ATGAATTCCGATGGACCCAA-3′ and 5′-GACCTCATCAGATACTGTGG-3′; For At1g68875, 5′-ATGACAGAACTCAAATGGAT-3′ and 5′-CTAGTTAGACTGTGGTGCCA-3′; For At1g02200, 5′-ATGGCCACAAAACCAGGAGT-3′ and 5′-GAATATCATGGAGAGAGAGG-3′; For At5g07550, 5′-ATGTTTGAGATTATTCAGGC-3′ and 5′-TTAGACGCCGGAACCTGCTG-3′.

### Real-time quantitative PCR

The *GmPHDs* were amplified with the following primers: for *GmPHD1*, 5′-ATGGACTCTCGCACGTATAA-3′ and 5′-GTGGTACTTCTTCAGCAGGT-3′; for *GmPHD2*, 5′-ATGGACGGTGGTGGAGTGAA-3′ and 5′-CCTTCCGCAGGTAAATTAAC-3′; for *GmPHD3*, 5′-ATGGAGGCGCTAAGTCGCTC-3′ and 5′-AAGCTCTGGAGGAACTTCTT-3′; for *GmPHD4*, 5′-ATGGAGGCAGGTTACAATCC-3′ and 5′-CAGGGGGCACCTCCTCAGCT-3′; for *GmPHD5*, 5′-ATGGAAGGAGTACCGCACCC-3′ and 5′-GCACTTCCTCAACAGGCAAA -3′; for GmPHD6, 5′-ATGGACAGTGGAGGACACTA-3′ and 5′- GGAACTTCTTCAGCAGGCAA -3′. Real-time PCR were performed on MJ PTC-200 Peltier Thermal Cycler based on previous protocol [7]. The results were analyzed using Opticon Monitor™ analysis software 3.1 (Bio-Rad). Each experiment had four replicates and was repeated twice.

### Localization of the GmPHD-GFP fusion proteins

The GmPHD coding sequences were amplified with the primers containing BamHI and SalI sites. The products were fused to the 5′ end of GFP to generate the pUC-GmPHDs-GFP constructs [60]. The pUC-GFP vector was used as control. These constructs were introduced into Arabidopsis protoplasts by PEG-mediated transfection. After culturing for 20 h, the fluorescence of GFP was visualized under fluorescence microscope.

### Dimerization of GmPHDs

Interaction of GmPHDs was investigated by co-transforming plasmids into the yeast strain YRG2 according to the manual (Stratagene). The pBD-GmPHDs and pAD-GmPHDs were made by insertion of the GmPHD coding region into pBD vector or pAD vector. Each of the six pBD-GmPHDs, together with each of the pAD-GmPHDs, was co-transformed into YRG2. The PHD finger sequences of each GmPHDs were amplified by PCR and then introduced into pBD vector to generate pBD-G1PHD, pBD-G2PHD, pBD-G3PHD, pBD-G4PHD, pBD-G5PHD and pBD-G6PHD. These plasmids, together with pAD-GmPHD6, were co-transformed into YRG2. The pBD-GmPHD5, pBD-G5N (amino acids 1 to 115), pBD-G5V (amino acids 116 to 197), pBD-G5PHD (amino acids 198–252), pBD-G5NV (amino acids 1 to 197) and pBD-G5PV (amino acids 116 to 252), together with pAD-GmPHD6, was also co-transformed into YRG2. pBD vector or pAD vector, together with the corresponding recombinant plasmids, was co-transformed into yeast cells as negative controls. The yeast transformants were plated onto SD-His/Trp/Leu plus 3-AT and the growth was examined. X-gal staining was performed to examine the *Lac*Z reporter gene expression [61].

### Transient assay for transcriptional activation/inhibition activity of GmPHDs in Arabidopsis protoplast system

Reporter plasmid 5XGAL4-LUC and internal control pPTRL (*Renilla reniformis* Luciferase driven by 35S promotor) were kindly provided by Dr. Masaru Ohme-Takagi. 5XGAL4-LUC contains five copies of GAL4 binding element and minimal TATA region of 35S promoter of Cauliflower Mosaic Virus (CaMV), located upstream of the firefly gene for luciferase [62]. Expression vector pRT-BD was constructed by insertion of the GAL4DBD coding region into pRT107 vector by Sac I/Xba I digestion. And the positive control (35S-BD-VP16) was constructed by insertion of VP16, a herpes simplex virus (HSV)-encoded transcriptional activator protein, into pRT-BD vector.

For effector plasmids used in Fig. 4a, the coding regions of GmPHDs were digested by BamHI/Sal I, and cloned into pRT-BD vector to generate 35S-BD-GmPHDs. For effector plasmids in Fig. 4bc, the coding regions of GmPHDs were digested by BamHI/Sal I, and cloned into pRT107 vector to generate 35S-GmPHDs, which will not compete for the GAL4 binding elements in reporter plasmid 5XGAL4-LUC when incubated with the 35S-BD-VP16. The truncated coding sequences of GmPHD2 were also cloned into pRT107 to obtain 35S-G2N (amino acids 1 to 117), 35S-G2V (amino acids 118 to 196), 35S-G2PHD (amino acids 197 to 253), 35S-G2NV (amino acids 1 to 196), and 35S-G2VP (amino acids 197 to 253). The Arabidopsis Dof23 (At4g21030) was used as a non-interactive control when incubated with 35S-BD-VP16.

The ratios in Fig. 4 indicate µg of each plasmid. The effectors, reporter and internal control were co-transfected into Arabidopsis protoplasts. After culturing for 16 h, Luciferase assays were performed with the Promega Dual-luciferase reporter assay system and the GloMax^TM^20-20 luminometer [7].

### Gel shift assay

The genes for GST-GmPHDs fusions and various domains of the GmPHD4 and GmPHD2 were cloned into pGEX-4T-1, and the proteins were expressed in E. coli (BL21) and purified according to the manual. A pair of oligonucleotides 5′-AATTCGGATCCGTGGAGGTGGAGGTGGAGGTGGAGGTGGAGGGTACCGAGCT-3 and 5′-CGGTACCCTCCACCTCCACCTCCACCTCCACCTCCACGGATCCG-3′ was synthesized. The two sequences contained five tandem repeats of “GTGGAG”. The double-stranded DNA was obtained by heating oligonucleotides at 70°C for 5 min and annealing at room temperature in 50 mM NaCl solution. Gel shift assay was performed as described [59].

### Generation of *GmPHD2*-transgenic plants and performance of the transgenic plants under salt-stress

The coding sequence of the *GmPHD2* was amplified by RT-PCR using primers 5′-GGAGGATCCATGGACTCTCGCACGTATAATCC-3′ and 5′-TGTGGTACCGGGCCGAGCTCTCTTGTTAC-3′, and cloned into the BamHI/KpnI sites of the pBIN438 under the control of CaMV 35S promoter. The homozygous T3 seeds were analyzed.

Seeds were plated on NaCl medium for germination tests. Plates were placed at 4°C for 3 d and then incubated in a growth chamber under continuous light at 23°C. Each value represents the average germination rate of 80–100 seeds with at least three replicates. For salt-stress tolerance tests, 5-day-old seedlings on MS agar medium were transferred on MS agar medium supplemented with 0, 50, 100, 150 and 200 mM NaCl respectively. The phenotypes were observed 2 weeks later. The seedlings under 150 mM NaCl treatment were further transferred into soil and grown for two weeks under normal conditions. Then the root length and height of plants were measured.

### Oxidative stress tolerance test and physiological parameters

Seeds were plated on the MS containing different concentrations of paraquat for germination tests. Each value represents the average germination rate of 80-100 seeds with at least three replicates.

Ten-day-old seedlings were transferred onto the MS plates containing 150 mM NaCl and maintained for 5 d. Plant leaves were cut and submerged in 1 mg/ml 3′,3′-diaminobenzidine (DAB) solution for 6 to 8 h and then fixed with solution of ethanol/lactic acid/glycerol (3∶1∶1, V/V/V). Brown color indicates presence of the hydrogen peroxide.

Measurement of peroxidase (POD) activity and relative electrolyte leakage were performed according to previous descriptions [63], [64].
